# A Young Female With Medium-Chain Acyl-CoA Dehydrogenase Deficiency (MCADD): A Case Report

**DOI:** 10.7759/cureus.36018

**Published:** 2023-03-11

**Authors:** Ibidapo Q Yusuf, Aadithiyavikram Venkatesan, Faith C Okafor, Athar Yasin, Samson O Oyibo

**Affiliations:** 1 General Medicine, Peterborough City Hospital, Peterborough, GBR; 2 Emergency Medicine, Peterborough City Hospital, Peterborough, GBR; 3 Diabetes and Endocrinology, Peterborough City Hospital, Peterborough, GBR

**Keywords:** medium-chain acyl-coa dehydrogenase deficiency, medium-chain acyl-coa dehydrogenase, acyl-coa dehydrogenase, metabolic crisis, emergency care plan, exercise training, sports injury surgery, rhabdomyolysis, hypoketotic hypoglycemia, mcadd

## Abstract

Medium-chain acyl-CoA dehydrogenase (MCAD) deficiency (MCADD) is a rare autosomal recessive inborn error of mitochondrial fatty acid oxidation. MCAD is essential for fatty acid β-oxidation during hepatic ketogenesis, which provides a major source of energy once hepatic glycogen stores are exhausted during extended fasting and periods of increased energy demand. The inability to metabolize these fatty acids results in hypoketotic hypoglycemia and the accumulation of toxic partially metabolized fatty acids. Intercurrent infection, extended fasting, excessive alcohol intake, vomiting, or diarrhea can lead to serious illness, including encephalopathy and even sudden death. Young people with MCADD are followed up on a regular basis by their metabolic disease specialist, and they are informed about risk factors as they advance through adolescence and adulthood. They should also carry along a written emergency management plan and relevant contact numbers. We describe a case of a 17-year-old female who attended her local emergency care center complaining of severe abdominal pain, vomiting, muscle ache, and poor oral intake. She was known to have MCADD; however, her emergency care plan had a date from eight years ago. She made a rapid recovery after receiving intravenous glucose and other therapies. The patient’s concerns and knowledge about MCADD were not fully appreciated at the initial stage due to the rare nature of the disease. This in combination with the absence of current notes on the system, an emergency care plan dated from eight years ago, and the need to obtain specialist advice led to a slight delay in commencing specific therapy. This case report serves as a reminder of the emergency presentation of young people with MCADD, emphasizing the importance of effective communication between the patient, their parents, and the treating clinicians, obtaining the emergency care plan and recommendations, and communicating with the metabolic disease specialist.

## Introduction

Medium-chain acyl-CoA dehydrogenase (MCAD) deficiency (MCADD) is a rare autosomal recessive inborn error of mitochondrial fatty acid oxidation characterized by a rapidly progressive metabolic crisis, often presenting with hypoketotic hypoglycemia, lethargy, vomiting, seizures, and coma [1,2]. MCADD is caused by mutations in the *ACADM* gene, which encodes the mitochondrial MCAD protein and is located on chromosome one (1p31) [1,2].

Fatty acid β-oxidation fuels hepatic ketogenesis, which provides a major source of energy once hepatic glycogen stores become depleted during extended fasting, and during periods of increased energy demand [2]. People with MCADD are unable to go through this process. Instead, they develop hypoketotic hypoglycemia and accumulate toxic partially metabolized fatty acids in their kidneys, liver, muscles, and brain. Intercurrent infection, prolonged fasting, excessive alcohol intake, vomiting, or diarrhea can lead to encephalopathy and even death. Symptomatic individuals should seek prompt medical assistance, which should include an intravenous 10% glucose infusion (2 ml/kg per hour) to maintain their blood glucose level above 5.0 mmol/L, to prevent hypoglycemia and the accumulation of toxic partially metabolized fatty acids. This treatment should be started even if the initial blood glucose level is in the normal range [3].

People with MCADD will be admitted to their local emergency care center in the event of illness for urgent treatment to prevent a metabolic crisis. We discuss a case of a young female with MCADD who presented to her local emergency care center for emergency treatment. This case report serves as a reminder of the emergency presentation of young people with MCADD, emphasizing the importance of effective communication between the patient, their parents, and the treating clinicians, obtaining the emergency care plan and recommendations, and communicating with the metabolic disease specialist.

## Case presentation

Medical history and demographics

A 17-year-old female presented with a four-day history of severe, intermittent abdominal pain and vomiting, and a one-day history of aching and mild weakness in her lower limbs. She had been managing little amounts of glucose drinks to maintain her calorie intake, but this became intolerable from the day before the presentation.

She had sustained a right knee injury during rugby training five months prior to this presentation and had been taking non-steroidal anti-inflammatory drugs (NSAIDs; ibuprofen and naproxen) for pain relief since then. She had no urinary symptoms, and her menstrual cycle was complete.

She was known to have MCADD since childhood and had regular follow up at a pediatric metabolic disease center. She was transferred to the adult metabolic disease team responsible for her region. She had an MCADD emergency care plan with her, but this was dated eight years ago. Aside from the NSAIDs listed above, she was not taking any additional medication. She was allergic to co-amoxiclav, dairy products, and coconut oils. She did not smoke or drink alcohol. She participated in rugby, horse riding, swimming, netball, and regular gymnasium workouts.

On examination, she had a Glasgow Coma Scale score of 15, with a blood pressure of 120/75 mmHg, heart rate of 66 beats per minute, respiratory rate of 18 breaths per minute, and oxygen saturation of 99% on room air. Abdominal examination revealed severe tenderness in the epigastric and umbilical area, but no guarding or rigidity. Bowel sounds were normal. She weighed 82 kg.

Investigations

Her initial bedside capillary blood glucose level was 5.3 mmol/L. Further investigation demonstrated normal full blood count, renal function, and liver function. Her C-reactive protein, venous pH, and venous lactate levels were normal. A pregnancy test was negative. The patient’s creatinine kinase and lactate dehydrogenase levels were slightly elevated, indicating mild rhabdomyolysis (Tables 1, 2). Electrocardiography was normal.

**Table 1:** Initial hematology results

Hematology blood test	Results	Reference range
White cell count (x 10^9^/L)	8.2	4-11
Hemoglobin (g/L)	143	115-165
Platelets (x 10^9^/L)	284	150-400
Neutrophils (x 10^9^/L)	6.4	1.8-7.7
Lymphocytes (x 10^9^/L)	1.2	1.4-4.8
Fibrinogen (g/L)	3.0	2.0-4.5
Prothrombin time (seconds)	13	9-16
Activated partial thromboplastin time (seconds)	29	24-36

**Table 2:** Initial biochemistry results Results on admission demonstrated slightly elevated creatinine kinase and lactate dehydrogenase levels.

Biochemistry blood test	Results	Reference range
C-reactive protein (mg/L)	<1	<5
Creatinine kinase (U/L)	515	25-200
Glucose (mmol/L)	5.6	3.9-7.0
Thyroid-stimulating hormone (mU/L)	3.62	0.3-4.2
Free thyroxine (pmol/L)	15.8	12-22
Sodium (mmol/L)	137	133-146
Creatinine (µmol/L)	74	45-84
Potassium (mmol/L)	4.5	3.5-5.3
Urea (mmol/L)	3.1	2.5-7.8
Chloride (mmol/L)	103	95-108
Anion gap (mmol/L)	6	4-16
Alkaline phosphatase (U/L)	81	30-130
Albumin (g/L)	49	35-50
Total bilirubin (µmol/L)	23	<21
Alanine transferase (U/L)	22	10-60
Amylase (U/L)	52	0-100
Adjusted calcium (mmol/L)	2.3	2.2-2.6
Inorganic phosphate (mmol/L)	0.71	0.8-1.5
Magnesium (mmol/L)	0.7	0.7-1.0
Lactate dehydrogenase (U/L)	262	<250
Ferritin (µg/L)	29	30-400
Vitamin B12 (pg/ml)	655	270-1132
Folate (µg/L)	7.3	>3
Venous pH	7.34	7.31-7.41
Venous bicarbonate (mmol/L)	28	23-30
Venous lactate (mmol/L)	1.07	0.2-1.8
Base excess (mmol/L)	1.2	-2 to +2

Treatment

We determined that the patient had NSAID-induced gastritis accompanied by mild rhabdomyolysis, based on the symptoms and biochemistry. As her blood glucose levels were in the normal range, an intravenous infusion of 0.9% sodium chloride was commenced for rehydration. She was also given an antiemetic to relieve her vomiting and an analgesic to relieve her pain. After consultation and evaluation of her emergency care plan, she was then commenced on specific therapy according to the emergency care plan: an intravenous infusion of 10% glucose (2 ml/kg per hour) to provide energy and prevent hypoglycemia. She was also given a proton-pump inhibitor to treat her gastritis. She had hourly monitoring of her capillary blood glucose levels while on the glucose infusion. She restarted her glucose polymer drinks and feeding once able to tolerate oral intake.

Outcome and follow-up

The patient made a rapid recovery and was discharged the following day on pantoprazole 40 mg daily and a liquid antacid for gastritis. Her serum creatinine kinase levels returned to normal three days after discharge. She was provided with an up-to-date emergency care plan, and a notification was put in her electronic records in case of future admissions.

## Discussion

We have presented a case of a young female known to have MCADD who presented to the emergency care center with the inability to keep up calorie intake because of NSAID-induced gastritis. She also had symptoms and biochemical features suggestive of mild rhabdomyolysis. Our hospital records for this patient relating to MCADD and her personal emergency care plan were dated eight years ago. Because her blood glucose level was normal, she was initially commenced on an intravenous 0.9% sodium chloride infusion. Once the emergency care plan was validated, the patient was commenced on an intravenous 10% glucose infusion, and she made a rapid recovery. The patient’s concerns and knowledge about MCADD were not fully appreciated at the initial stage due to the rare nature of the condition. This in combination with the absence of current notes on the system, an emergency care plan dated from eight years ago, and the need to obtain specialist advice led to a slight delay in starting specific therapy.

The global birth prevalence of MCADD is around 1/14,000 [1]. Affected children are monitored on a regular basis by their metabolic disease pediatrician. As they grow into teens and adults, they are educated about the risk factors and potential dangers associated with hypoglycemia. Risk factors include weight-reducing regimens, competitive sports, and surgery as well as pregnancy and delivery, and the use of alcohol and illicit drugs. People with MCADD should carry along a written emergency management plan and relevant contact numbers [4]. With proper care, there is no reason why people with MCADD cannot live a normal, healthy, and active life.

Rhabdomyolysis has been reported as a presenting feature both at diagnosis and during a metabolic crisis in patients with fatty acid oxidation disorders, namely, MCADD and very long-chain acyl-CoA dehydrogenase deficiency (VLCADD) [4,5]. Rhabdomyolysis is characterized by acute and often severe skeletal muscle damage resulting in the release of intracellular muscle components into the bloodstream frequently resulting in myoglobinuria and, in severe cases, acute renal failure [6,7]. Causes of rhabdomyolysis can be divided into acquired and genetic. The diverse etiologies share a common final pathway, involving increased intracellular free ionized calcium and muscle cell death through the activation of several detrimental mechanisms such as enzymatic activation and prolonged muscle fiber contraction [6,7]. Patients present with muscle aches, weakness, and, in some cases, dark urine. Triggers for rhabdomyolysis in patients with MCADD include fasting, extreme exercise, alcohol, and illicit drugs [7]. Our patient had symptoms and biochemical features of mild rhabdomyolysis, which could have got worse if emergency treatment was not promptly commenced.

Living with MCADD is challenging for both young people and their parents. Parental challenges include preventing and managing illness, dietary monitoring, schooling, excursions, and exercise. These concerns and anxieties increase as the child gets older [8]. Young people with MCADD perceive a burden of responsibility that they must maintain appropriate energy input to stay safe [9]. The importance of self-management and continued support for young people with MCADD and their parents cannot be overemphasized.

People with MCADD may require interim follow-up, medication advice, prescriptions, and blood tests at their local clinic, especially if they have other comorbidities. The rare coexistence of MCADD and type 1 diabetes has been described in a case report. Where the goal of therapy is to achieve optimal glycemic control to reduce the risk of long-term complications, there is an increased risk of insulin-induced hypoglycemia, which can be catastrophic in the presence of MCADD [10]. We have previously described the challenges involved in managing a patient with coexisting MCADD, type 1 diabetes, and pregnancy, where the blood glucose control was kept even tighter for optimum fetal development and pregnancy outcome [11]. The importance of continued liaison between the local hospital team and the metabolic disease specialist cannot be overemphasized.

## Conclusions

People with inborn errors of metabolism, such as MCADD, are at risk of metabolic crisis and will attend their local emergency care center in the event of illness. This case report serves as a reminder of the emergency presentation of young people with MCADD, emphasizing the importance of effective communication between the patient, their parents, and the treating clinicians, obtaining the emergency care plan and recommendations, and communicating with the metabolic disease specialist.

## References

[1] (2023). The portal for rare diseases and orphan drugs (Orphanet). Medium chain acyl-CoA dehydrogenase deficiency. https://www.orpha.net/consor/cgi-bin/OC_Exp.php?Lng=GB&Expert=42.

[2] Merritt JL 2nd, Chang IJ (2023). Medium-chain acyl-coenzyme A dehydrogenase deficiency. GeneReviews.

[3] (2023). British Inherited Metabolic Disease Group (BIMDG). Adult emergency management: medium chain fat oxidation disorders. https://www.bimdg.org.uk/store/guidelines/ADULT_MCAD-rev_2015_566641_09012016.pdf.

[4] Schatz UA, Ensenauer R (2010). The clinical manifestation of MCAD deficiency: challenges towards adulthood in the screened population. J Inherit Metab Dis.

[5] Fatehi F, Okhovat AA, Nilipour Y (2020). Adult-onset very-long-chain acyl-CoA dehydrogenase deficiency (VLCADD). Eur J Neurol.

[6] Scalco RS, Gardiner AR, Pitceathly RD (2015). Rhabdomyolysis: a genetic perspective. Orphanet J Rare Dis.

[7] Hannah-Shmouni F, McLeod K, Sirrs S (2012). Recurrent exercise-induced rhabdomyolysis. CMAJ.

[8] Piercy H, Machaczek K, Ali P, Yap S (2017). Parental experiences of raising a child with medium chain acyl-COA dehydrogenase deficiency. Glob Qual Nurs Res.

[9] Piercy H, Nutting C, Yap S (2021). “It’s just always eating”: the experiences of young people growing up medium chain acyl-CoA dehydrogenase deficiency. Glob Qual Nurs Res.

[10] Afreh-Mensah D, Agwu JC (2021). Coexistence of medium chain acyl-CoA dehydrogenase deficiency (MCADD) and type 1 diabetes (T1D): a management challenge. BMJ Case Rep.

[11] Wilson D, Brown A, Gumma AD, Oyibo SO (2017). Blood glucose control in a pregnant female with type 1 diabetes and medium-chain acyl-CoA dehydrogenase deficiency (MCADD). Endocr Abstr.

